# High SEPT9_i1 Protein Expression Is Associated with High-Grade Prostate Cancers

**DOI:** 10.1371/journal.pone.0124251

**Published:** 2015-04-21

**Authors:** Roni Gilad, Karen Meir, Ilan Stein, Larissa German, Eli Pikarsky, Nicola J. Mabjeesh

**Affiliations:** 1 Department of Urology, Tel Aviv Sourasky Medical Center, Sackler Faculty of Medicine, Tel Aviv University, Tel Aviv, Israel; 2 Department of Pathology, The Hebrew University-Hadassah Medical School, Jerusalem, Israel; Innsbruck Medical University, AUSTRIA

## Abstract

Septins are a family of GTP-binding cytoskeleton proteins expressed in many solid tumors. Septin 9 (*SEPT9*) in particular was found overexpressed in diverse carcinomas. Herein, we studied the expression of SEPT9 isoform 1 protein (SEPT9_i1) in human prostate cancer specimens. We utilized immunohistochemical staining to study the expression of SEPT9_i1 protein. Staining level was analyzed in association with clinical characteristics and the pathological Gleason grade and score. Fifty human prostate cancer specimens (42 primary tumors and 8 metastatic lesions) were stained by SEPT9_i1 antibody and analyzed. SEPT9_i1 protein was expressed in prostate cancer cells but absent in normal epithelial cells. The intensity of staining was correlated proportionally to pretreatment prostate-specific antigen (PSA) blood levels and Gleason score (*P* < 0.05). SEPT9_i1 was highly expressed in all metastatic lesions. A significant assocation between SEPT9_i1 expression and high Gleason score on multivariate linear regression analysis was found. We conclude that SEPT9_i1 is expressed in high-grade prostate tumors suggesting it has a significant role in prostate tumorigenesis and that it could serve as a molecular marker for prostate tumor progression.

## Introduction

Prostate cancer is second to lung cancer in incidence worldwide and is the second most common cancer, and one of the leading causes of cancer deaths among Western men [1]. The primary risk factors for prostate cancer are age and family history. Prostate cancer becomes more common with advancing age affecting men aged 50 or more [2]. Prostate adenocarcinoma metastasizes mainly to the bones and less commonly to lymph nodes, and may locally advance to invade neighboring organs. Current treatments include hormonal therapy, immunotherapy and chemotherapy, yet there is no curative treatment for metastatic prostate cancer [3, 4]. Therefore, novel strategies for treatment of prostate cancer and identification and characterization of new molecular targets such as interleukin-6 are essential [5].

Septins are a family of GTP-binding and filament forming proteins, first described in *Saccharomyces cerevisiae* in a screen for genes that regulate the budding process [6]. Since then, septins have been identified in many other eukaryotes, ranging from fungi to humans [7–9] with a notable absence in plants [10]. Many septin isoforms are abnormally expressed in carcinomas [11], and altered levels of septin expression strongly correlate with tumorigenic phenotypes such as increased cell growth, motility, invasiveness, and resistance to microtubule-disrupting reagents [12, 13].

We had previously identified SEPT9_i1, a product of transcript SEPT9_v1 that encodes isoform 1 with the largest N-terminal extension, as a positive regulator in the hypoxic pathway [14]. SEPT9_i1 interacts with hypoxia-inducible factor 1α (HIF-1α), the oxygen-regulated subunit of HIF-1, which is a key regulator of the hypoxic response pathway [15]. The interaction is specific to HIF-1α, but not to HIF-2α, and it increases HIF-1α protein stability as well as HIF-1 transcriptional activity, leading to enhanced proliferation, tumor growth and angiogenesis. HIF-1 is a transcription factor that regulates the responses and cellular adaptation to hypoxia driving transcription of many genes that are important for adaptation and survival under hypoxia [14]. Among these genes are glycolytic enzymes, the glucose transporters Glut-1 and Glut-3, endothelin-1,vascular endothelial growth factor, carbonic anhydrase IX, and erythropoietin [16]. Immunohistochemical analyses revealed that HIF-1α is overexpressed in many human cancers [17]. Furthermore, increased HIF-1 activity is often associated with increased tumor aggressiveness, therapy resistance, and mortality [18].

Our previous studies in various prostate cell lines and xenografts showed that SEPT9_v1 mRNA is highly expressed in human prostate cancer samples compared with normal prostate tissue [15]. In the present study, we determined the expression of SEPT9_i1 protein in primary human prostate cancer tissues using immunohistochemistry and correlated its expression with clinicopathologic characteristics.

## Materials and Methods

### Tissue samples

The institutional review board of the Hebrew University-Hadassah Medical Center approved this study (IRB protocol 0500-12-HMO). Archival material prior to the year 2000 was approved for use by the institutional review board of the Hebrew University-Hadassah Medical Center, with a waiver of informed consent, in accordance with the State of Israel Law of Genetic Information, 2002. All archival specimens used in this study were obtained from the institutional biorepository at Hadassah Medical Center. All study specimens were prior to year 2000 and all were de-identified. This was a retrospective study on a series of 50 paraffin-embedded prostate cancer samples: 38 from radical prostatectomy, 6 from radical cystoprostatectomy and 6 from transurethral resection of prostate (TUR-P). Of this collection, eight specimens were excluded from the study due to technical problems in tissue processing leaving 42 primary tumors at different stages for final evaluation (Table 1). Eight additional metastatic lesions from 8 different patients, bone marrow (3), lymph nodes (2) and bone (3) were analyzed separately. One sample from each patient was analyzed and none of the patients received neoadjuvant therapy.

**Table 1:** Main characteristics of study participants.

Variables		Total patients (n = 42)
Age, years		
	mean (SD)	64.9 (6.9)
	median (range)	65.0 (51–83)
	95% CI	62.7–67.1
PSA level, ng/ml		
	mean (SD)	6.9 (4.2)
	median (range)	5.9 (1.6–26.0)
	95% CI	5.4–8.4
Gleason score, n (%)		
	<7	8 (19.0)
	= 7	20 (47.6)
	>7	13 (31.0)
	NA	1 (2.4)
T stage, n (%)		
	T1-2	23 (54.8)
	T3	14 (33.3)
	NA	5 (11.9)

PSA, prostate-specific antigen; CI, confidence interval; NA, not available.

### Immunohistochemistry

**Fig 1:**
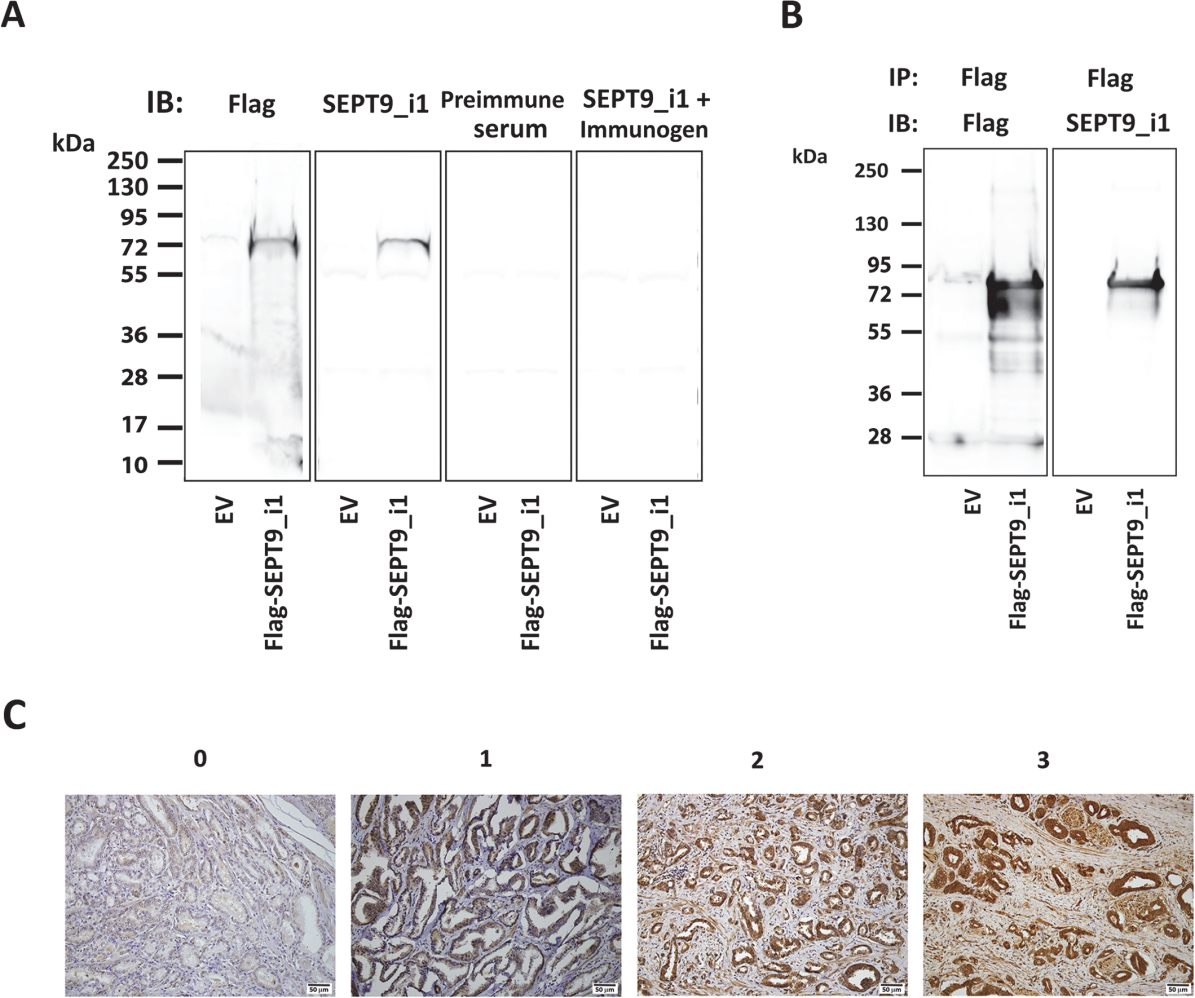
Characterization of SEPT9_i1 antibodies and scoring of SEPT9_i1 staining intensity. HEK-293T embryonic human kidney cells were transiently transfected with Flag-SEPT9_i1 construct or empty vector (EV). (A) Whole cellular extracts were prepared and analyzed by 4–20% SDS-PAGE and immunoblotting (IB) with antibodies to Flag (1:2000), SEPT9_i1 (1:3000), preimmune serum (1:3000) or SEPT9_i1 antibody (1:3000) pre-incubated with 10 μM of the immunogen peptide for 4 hours. (B) The same cellular extracts were subjected to immunoprecipitation (IP) using anti-Flag antibody and the immuneprecipitates were subjected to 4–15% SDS-PAGE and then immunoblotted with anti-Flag or anti-SEPT9_i1 antibodies. (C) Representative SEPT9_i1 staining in human prostate cancer specimens. Score 0: no SEPT9_i1 staining, 1: low SEPT9_i1 staining 2: medium SEPT9_i1 staining and 3: high SEPT9_i1 staining. Magnification x200, scale bar 50 μm.

A rabbit polyclonal antibody directed to the N-terminus of SEPT9_i1 was previously produced [15] and further characterized (Fig 1A and 1B). Immunohistochemical staining was performed using the same protocol for all tissues. Briefly, 4 μm formalin-fixed paraffin-embedded sections were deparaffinized, rehydrated and antigen retrieval was performed in 25mM citrate buffer pH 6 by heating to 125°C for 3 min in decloaking chamber (Biocare Medical). Sections were incubated with anti-SEPT9_i1 antibody at 1:2000 dilution overnight at 4°C.

Anti-rabbit HRP-conjugated antibody (Vector Labs) was used to detect the primary antibody. Slides were developed with diaminobenzidine (DAB) and counterstained with hematoxylin.

### Quantitative evaluation of immunostaining and scoring

All slides were reviewed by two independent investigators (RG and EP) who were blinded to all clinical and pathological data. Intensity of staining was scored as 0 (no expression), 1 (weak), 2 (moderate) and 3 (strong). We also performed computerized image analysis using an ARIOL-SL50 automated scanning microscope and image analysis system (Applied Imaging) to evaluate the intensity of specimen staining, essentially as described [19]. Slides were scanned and all tumor areas were marked. From each tumor 10 separate fields, randomly selected, were analyzed and normalized according to the manufacturers setting (Hersight module). The results of the analysis are presented as normalized mean intensity as calculated by the ARIOL-SL50 software.

### Transient transfection, protein extraction, immunoprecipitation assays, and immunoblot analysis

Human embryonic kidney (HEK 293T) cells were seeded at 70% confluence in 6 cm plates and were transfected with 3 μg of a plasmid expressing Flag-SEPT9_i1 protein or empty vector (EV), using GenePorter transfection reagent (Gene Therapy Systems, Inc., San Diego, CA) as previously described [15]. Whole cell extracts were prepared and protein concentrations were determined using a bicinchoninic acid protein assay kit (Pierce, Rockford, IL) as previously described [15]. Immunoprecipitation was carried out using Ezview Red ANTI-FLAG Affinity gel (Sigma-Aldrich Co., St. Louis, MO), and by following the manufacturer’s instructions. Proteins were then analyzed by SDS-PAGE and immunoblotting with antibodies as displayed in the Figs. basically as previously described [15]. Primary antibodies were rabbit polyclonal antibody to SEPT9_i1, which was previously produced [15] and its corresponding pre-immune serum or to Flag (Sigma-Aldrich, St. Louis, MO). Secondary antibody was horseradish peroxidase conjugated Goat anti-mouse or rabbit (Jackson ImmunoResearch, West Grove, PA).

### Statistical analyses

Descriptive statistics of the study sample were used to summarize participant characteristics. Bivariate Spearman's correlation analyses were used to assess the relationship between "patients' characteristics" and mean. In a multivariate linear regression analysis, the outcome was mean SEPT9_i1 staining intensity. The independent variables included age and Gleason score. Adjusted β and SE were computed. The best model was determined considering adjusted R^2^. All tests were two-tailed and statistical significance was defined as *P* value<0.05. The analyses were performed using the PASW Statistics 17.0 for Windows.

## Results

The main characteristics of participants whose samples were used in the study are described in Table 1. Median age was 65 years with median PSA of 5.9 ng/ml and the majority had Gleason score ≥ 7. We stained all patients’ samples using a SEPT9_i1-specific antibody [15]. We first characterized the specificity of SEPT9_i1 antibody. Flag-tagged SEPT9_i1 construct was transiently expressed in HEK-293T cells and subjected to immunoblotting with antibodies to Flag, SEPT9_i1 and SEPT9_i1 in the presence of the immunogen peptide [15] as well as with the preimmune serum (Fig 1A). Flag and SEPT9_i1 antibodies reacted with one 70 kDa band corresponding to Flag-SEPT9_i1 protein while the preimmune serum did not show any reactivity (Fig 1A). Furthermore, the immunoreactivity of SEPT9_i1antibody was almost completely abolished when the immunogen peptide was added (Fig 1A). In addition, the immunoprecipitated species by Flag antibody was also recognized by anti-SEPT9_i1antibodies (Fig 1B). These results further conform the specificity of the produced anti-SEPT9_i1antibodies.

**Fig 2:**
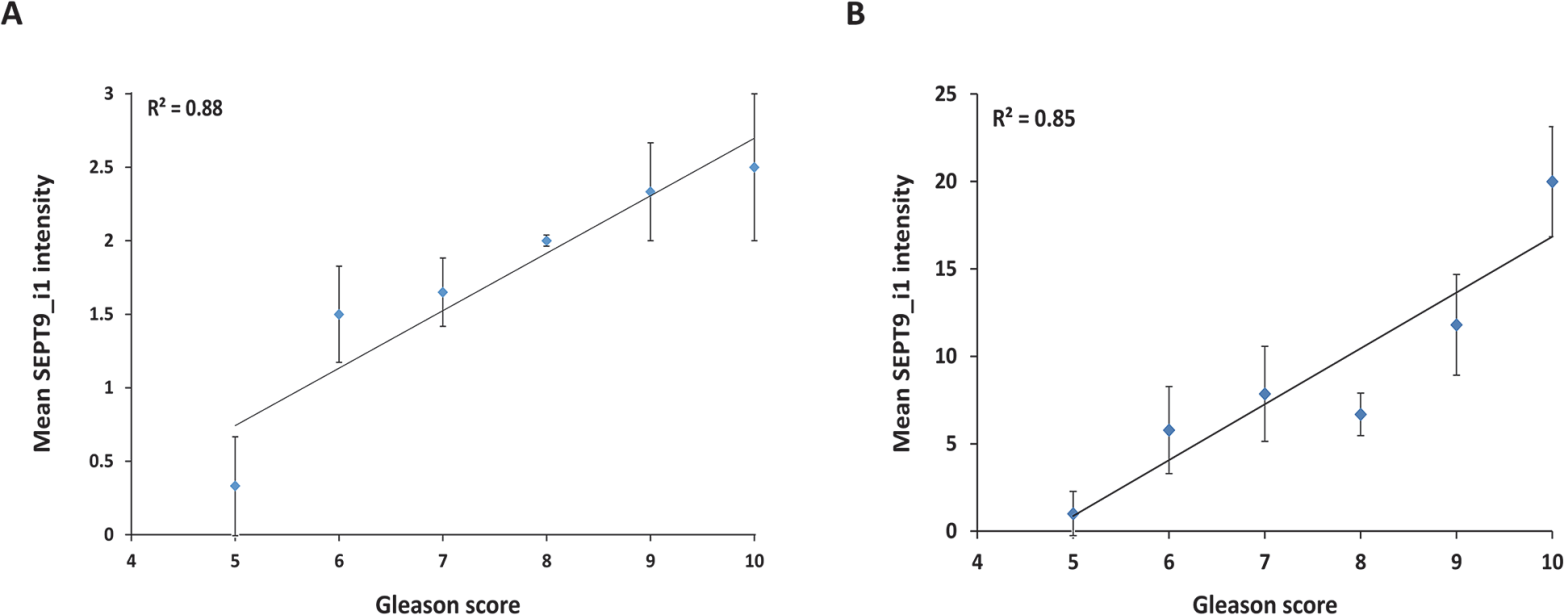
Gleason score correlation with SEPT9_i1 staining intensity. Mean SEPT9_i1 staining intensity ± SE of each Gleason score group (score 5: 3 patients, score 6: 8 patients, score 7: 19 patients, score 8: 3 patients, score 9: 6 patients and score 10: 2 patients) was calculated using either visual scoring (A) or automated image analysis with the ARIOL-SL50 system (B).

In the non-neoplastic prostate we observed prominent SEPT9_i1 cytoplasmic staining in most basal cells while luminal cells were negative (Fig 1C). Stromal cells staining in both neoplastic and non neoplastic regions was variable. Tumor cells showed variable staining intensity, which tended to be cytoplasmic however, in some cases was nuclear. The staining intensity of tumor cells was in some cases variable. In these cases the score was assigned based on the most abundant staining intensity. Each sample was then scored from 0 to 3 for expression level of SEPT9_i1 (Fig 1C). We then correlated SEPT9_i1 staining intensity with clinical characteristics and found strong and significant correlation between SEPT9_i1 expression levels and Gleason score (*P* = 0.01, Table 2). On multivariate linear regression analysis for the association between patients’ characteristics and mean SEPT9_i1 staining intensity, Gleason score was the only independent predictive factor (RR 2.0, 95% CI 0.15–3.85, *P* = 0.035). We stratified SEPT9_i1 staining intensity either by using visual scoring (Fig 2A) or by computerized image analysis with an automated image analysis system (ARIOL-SL50) (Fig 2B) according to Gleason score. There was significantly higher level of SEPT9_i1 staining as the Gleason score was higher (Fig 2).

**Table 2:** Correlation between "patients' characteristics" and SEPT9_i1 staining.

Variable	Age	Gleason score	T stage	PSA
Coefficient	-0.28	0.40	0.32	0.32
*P* value	0.08	0.01	0.06	0.04

PSA, prostate-specific antigen.

**Fig 3:**
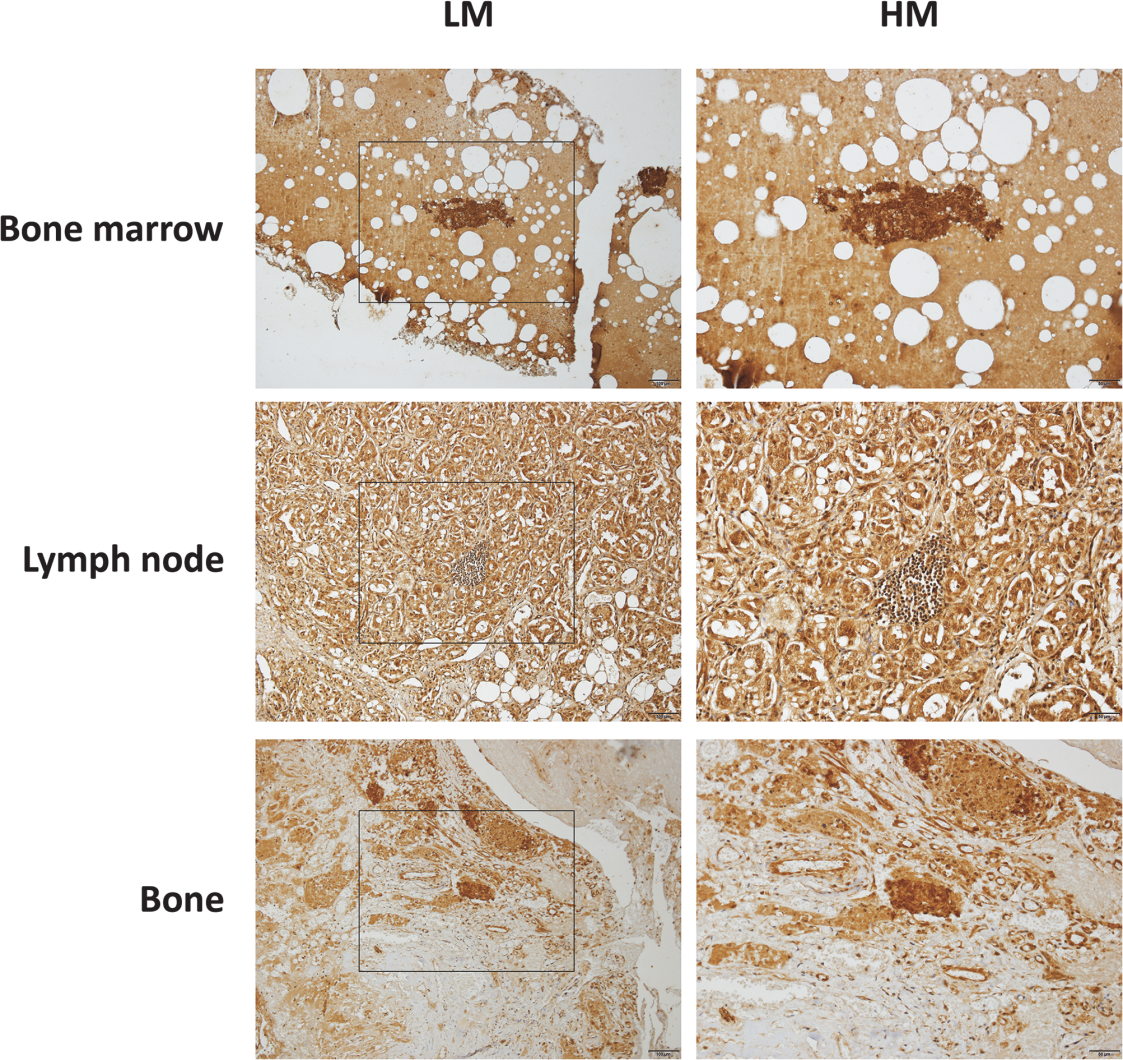
SEPT9_i1 staining in prostate cancer metastases. Metastatic prostate cancer lesions from bone marrow, lymph node and bone were immunostained with SEPT9_i1. Left panels are low (x100) magnification (LM) (scale bar 100 μm) and right panel are high (x200) magnification (HM) (scale bar 50 μm). Note high level of SEPT9_i1 staining in all metastases.

We next analyzed metastatic prostate cancer specimens from different sites including bone (3 samples), bone marrow (3 samples) and lymph nodes (2 samples). All metastatic lesions were strongly positive for SEPT9_i1 staining (Fig 3). Altogether, high expression of SEPT9_i1 in high-grade prostate cancer and in prostate cancer metastases suggests that SEPT9_i1 is a candidate marker for tumor progression.

## Discussion

High levels of SEPT9_i1 expression were observed in several malignancies including leukemia [20] and breast cancer [11]. In this study, we showed that SEPT9_i1 protein expression is significantly associated with Gleason score (Figs 1 and 2 and Table 2). Until these days, the Gleason grading and scoring system remains one of the most powerful prognostic factors for prostate cancer [21]. Most importantly, a significant intense SEPT9_i1 staining was seen in metastases of prostate adenocarcinoma to various sites (Fig 3). These results suggest that SEPT9_i1 protein expression strongly correlates with tumor aggressiveness and progression. Similarly, Stanbery et al. showed that high SEPT9_i1 protein expression in head and neck squamous cell carcinomas was associated with poor clinical outcomes [22]. Furthermore, they found that high expression of SEPT9_i1 correlated with both advanced T and N stage [22]. Collectively, these clinical data support previous studies showing the role of SEPT9_i1 in oncogenesis [15, 23–25].

Active surveillance is one of the accepted approaches utilized to reduce the treatment of patients with low-risk prostate cancer. Such patients are monitored with digital rectal physical examination, periodic blood PSA assessments and repeat prostate biopsies, and in some recent protocols selective imaging using multiparametric endorectal MRI [26]. Treatment is then offered to those with signs of progression. Unfortunately, multiple reports show that a substantial portion (about 30%) of men with low-risk prostate cancer are misclassified and need to receive immediate definitive treatment [27]. However, currently there is no accurate model to predict which of those patients with low-risk disease should be treated immediately before they progress during surveillance [28]. Since SEPT9_i1 protein expression is strongly correlated with tumor progression and metastasis, we propose SEPT9_i1 as a novel marker that may distinguish between low-risk versus higher-risk prostate cancer for better patients’ selection in active surveillance programs. Of course, further studies are warranted to test this hypothesis.

One of the limitations of our study is the lack of long-term clinical outcome for comparison with pathological staining intensity of SEPT9_i1. Another limitation is the relatively small number of patients in our cohort although it was sufficient to reach statistical significance.

## Conclusions

Altogether, our data clearly shows that SEPT9_i1 expression in prostate cancer is strongly associated with the most powerful pathological predictive factor, the Gleason score. Furthermore, high SEPT9_i1 expression levels were detected in all prostate adenocarcinoma metastases suggesting that SEPT9_i1 has a role in tumor invasiveness and progression. Further studies are needed to determine the applicability of SEPT9_i1 immunostaining in the clinical setting.

## References

[1] Ferlay J, Shin HR, Bray F, Forman D, Mathers C, Parkin DM. Estimates of worldwide burden of cancer in 2008: GLOBOCAN 2008. Int J Cancer. 2008;127:2893–917. 10.1002/ijc.25516 21351269

[2] Lindstrom S, Schumacher FR, Cox D, Travis RC, Albanes D, Allen NE, et al Common genetic variants in prostate cancer risk prediction—results from the NCI Breast and Prostate Cancer Cohort Consortium (BPC3). Cancer Epidemiol Biomarkers Prev. 2012;21:437–44. 10.1158/1055-9965.EPI-11-1038 22237985 PMC3318963

[3] Lechleider RJ, Arlen PM, Tsang KY, Steinberg SM, Yokokawa J, Cereda V, et al Safety and immunologic response of a viral vaccine to prostate-specific antigen in combination with radiation therapy when metronomic-dose interleukin 2 is used as an adjuvant. Clin Cancer Res. 2008;14:5284–91. 10.1158/1078-0432.CCR-07-5162 18698048 PMC2639763

[4] Nesslinger NJ, Ng A, Tsang KY, Ferrara T, Schlom J, Gulley JL, et al A viral vaccine encoding prostate-specific antigen induces antigen spreading to a common set of self-proteins in prostate cancer patients. Clin Cancer Res. 2010;16:4046–56. 10.1158/1078-0432.CCR-10-0948 20562209 PMC2912964

[5] Culig Z. New insights into the role of interleukin-6 in human prostate cancer. The Journal of urology. 2009;182:1255–6. 10.1016/j.juro.2009.07.073 19683299

[6] Hartwell LH. Genetic control of the cell division cycle in yeast. IV. Genes controlling bud emergence and cytokinesis. Exp Cell Res. 1971;69:265–76. 4950437 10.1016/0014-4827(71)90223-0

[7] Kinoshita M. The septins. Genome Biol. 2003;4:236 14611653 10.1186/gb-2003-4-11-236 PMC329110

[8] Mostowy S, Bi E, Fuchtbauer EM, Goryachev AB, Montagna C, Nagata K, et al Highlight: the 5th International Workshop on Septin Biology. Biol Chem. 2014;395:119–21. 10.1515/hsz-2013-0291 24334412

[9] Sandrock K, Bartsch I, Blaser S, Busse A, Busse E, Zieger B. Characterization of human septin interactions. Biol Chem. 2011;392:751–61. 10.1515/BC.2011.081 21767235

[10] Oh Y, Bi E. Septin structure and function in yeast and beyond. Trends Cell Biol. 2011;21:141–8. 10.1016/j.tcb.2010.11.006 21177106 PMC3073566

[11] Connolly D, Abdesselam I, Verdier-Pinard P, Montagna C. Septin roles in tumorigenesis. Biol Chem. 2011;392:725–38. 10.1515/BC.2011.073 21740328

[12] Amir S, Mabjeesh NJ. SEPT9_V1 protein expression is associated with human cancer cell resistance to microtubule-disrupting agents. Cancer Biol Ther. 2007;6:1926–31. 18075300 10.4161/cbt.6.12.4971

[13] Chacko AD, McDade SS, Chanduloy S, Church SW, Kennedy R, Price J, et al Expression of the SEPT9_i4 isoform confers resistance to microtubule-interacting drugs. Cell Oncol (Dordr). 2012;35:85–93. 10.1007/s13402-011-0066-0 22278362 PMC12995059

[14] Semenza GL. HIF-1 mediates metabolic responses to intratumoral hypoxia and oncogenic mutations. J Clin Invest. 2013;123:3664–71. 10.1172/JCI67230 23999440 PMC3754249

[15] Amir S, Wang R, Matzkin H, Simons JW, Mabjeesh NJ. MSF-A interacts with hypoxia-inducible factor-1alpha and augments hypoxia-inducible factor transcriptional activation to affect tumorigenicity and angiogenesis. Cancer Res. 2006;66:856–66. 16424018 10.1158/0008-5472.CAN-05-2738

[16] Semenza GL. Regulation of oxygen homeostasis by hypoxia-inducible factor 1. Physiology (Bethesda). 2009;24:97–106. 10.1152/physiol.00045.2008 19364912

[17] Mabjeesh NJ, Amir S. Hypoxia-inducible factor (HIF) in human tumorigenesis. Histol Histopathol. 2007;22:559–72. 17330811 10.14670/HH-22.559

[18] Quintero M, Mackenzie N, Brennan PA. Hypoxia-inducible factor 1 (HIF-1) in cancer. Eur J Surg Oncol. 2004;30:465–8. 15135470 10.1016/j.ejso.2004.03.008

[19] Yutkin V, Pode D, Pikarsky E, Mandelboim O. The expression level of ligands for natural killer cell receptors predicts response to bacillus Calmette-Guerin therapy: a pilot study. The Journal of urology. 2007;178:2660–4. 17945285 10.1016/j.juro.2007.07.118

[20] Santos J, Cerveira N, Bizarro S, Ribeiro FR, Correia C, Torres L, et al Expression pattern of the septin gene family in acute myeloid leukemias with and without MLL-SEPT fusion genes. Leukemia research. 2010;34:615–21. 10.1016/j.leukres.2009.08.018 19748670

[21] Epstein JI. An update of the Gleason grading system. The Journal of urology. 2010;183:433–40. 10.1016/j.juro.2009.10.046 20006878

[22] Stanbery L, D'Silva NJ, Lee JS, Bradford CR, Carey TE, Prince ME, et al High SEPT9_v1 Expression Is Associated with Poor Clinical Outcomes in Head and Neck Squamous Cell Carcinoma. Transl Oncol. 2010;3:239–45. 20689765 10.1593/tlo.10109 PMC2915415

[23] Amir S, Golan M, Mabjeesh NJ. Targeted knockdown of SEPT9_v1 inhibits tumor growth and angiogenesis of human prostate cancer cells concomitant with disruption of hypoxia-inducible factor-1 pathway. Mol Cancer Res. 2010;8:643–52. 10.1158/1541-7786.MCR-09-0497 20407014

[24] Chacko AD, Hyland PL, McDade SS, Hamilton PW, Russell SH, Hall PA. SEPT9_v4 expression induces morphological change, increased motility and disturbed polarity. J Pathol. 2005;206:458–65. 15902694 10.1002/path.1794

[25] Gonzalez ME, Peterson EA, Privette LM, Loffreda-Wren JL, Kalikin LM, Petty EM. High SEPT9_v1 expression in human breast cancer cells is associated with oncogenic phenotypes. Cancer Res. 2007;67:8554–64. 17875694 10.1158/0008-5472.CAN-07-1474

[26] Mullins JK, Bonekamp D, Landis P, Begum H, Partin AW, Epstein JI, et al Multiparametric magnetic resonance imaging findings in men with low-risk prostate cancer followed using active surveillance. Bju International. 2013;111:1037–45. 10.1111/j.1464-410X.2012.11641.x 23464904 PMC3978179

[27] van den Bergh RC, Ahmed HU, Bangma CH, Cooperberg MR, Villers A, Parker CC. Novel tools to improve patient selection and monitoring on active surveillance for low-risk prostate cancer: a systematic review. European urology. 2014;65:1023–31. 10.1016/j.eururo.2014.01.027 24491309

[28] Welty CJ, Carroll PR. The ongoing need for improved risk stratification and monitoring for those on active surveillance for early stage prostate cancer. European urology. 2014;65:1032–3. 10.1016/j.eururo.2014.02.044 24636678

